# Effect of an activity wristband-based intermittent teaching unit in Physical Education on studentsʼ physical activity and its psychological mediators: a cluster-randomized controlled trial. School-fit study

**DOI:** 10.3389/fpsyg.2023.1228925

**Published:** 2023-09-20

**Authors:** Santiago Guijarro-Romero, Daniel Mayorga-Vega, Carolina Casado-Robles, Jesús Viciana

**Affiliations:** ^1^Department of Didactic of Musical, Plastic and Corporal Expression, University of Valladolid, Valladolid, Spain; ^2^Departamento de Didáctica de las Lenguas, las Artes y el Deporte, Facultad de Ciencias de la Educación, Universidad de Málaga, Málaga, Spain; ^3^Department of Physical Education and Sport, University of Granada, Granada, Spain

**Keywords:** innovative program, wrist-worn wearables, autonomy support, motivation, physical activity levels, trans-contextual model, high school students

## Abstract

**Objective:**

The main objective of the present study was to examine the effects of an intermittent teaching unit based on the use of activity wristbands and behavior modification strategies on high school studentsʼ perceptions of autonomy support, satisfaction of basic psychological needs, motivation toward Physical Education and physical activity, intention to be physically active, and habitual physical activity levels.

**Materials and methods:**

An initial sample of 353 high school students (final sample = 175; 45.7% females; mean age = 13.3 ± 1.2 years) from two public high schools were cluster-randomly assigned into the intermittent (IG, *n* = 100) and control (CG, *n* = 75) groups. The IG performed an intermittent teaching unit twice a week for eight weeks. Specifically, the last 15 min of each lesson were used. As the main strategy to promote the practice of regular physical activity, students wore an activity wristband the whole day during the intervention period. Additionally, other behavior modification strategies were also applied (e.g., educational counseling, physical activity goals or reminders). Regarding the CG, during the intervention period (i.e,, the eight weeks that took place the intermittent teaching unit) they also performed two Physical Education sessions, but without using activity wristbands or other behavior modification strategies. Before and after the intervention, as well as at the end of the follow-up period (six weeks), students’ physical activity practice mediators and physical activity levels were measured by validated questionnaires.

**Results:**

The Multilevel Linear Model results showed that the IG students statistically significantly improved cognitive and procedural autonomy support from pre- to post-intervention (*p* < 0.05). They also statistically significantly improved autonomy and relatedness basic psychological needs, and autonomous motivation toward physical activity scores from post-intervention to follow-up (*p* < 0.05). Moreover, the results showed that the IG students statistically significantly improved habitual physical activity scores from pre- to post-intervention, and from post-intervention to follow-up (*p* < 0.05).

**Conclusion:**

The intermittent teaching unit based on the use of activity wristbands and other behavior modification strategies was effective for improving studentsʼ autonomy support and habitual physical activity levels, but not the rest of physical activity practice mediators.

**Clinical trial registration:**

https://register.clinicaltrials.gov/, ID: NCT05949463.

## 1. Introduction

Health benefits of regular physical activity (PA) in adolescents are well known (World Health Organization, 2020). The World Health Organization (2020) recommends that adolescents should do at least an average of 60 min per day of moderate-to-vigorous PA (MVPA) across the week. However, worldwide more than 80% of adolescents do not meet this recommendation (Guthold et al., 2020). Therefore, the promotion of adequate PA levels among adolescents is an important public health priority, which can take place in different settings of daily life (World Health Organization, 2018). Among others, the Physical Education (PE) subject plays an essential role in adolescents’ PA promotion (Association for Physical Education, 2020; World Health Organization, 2020), because it has been shown to have positive effects on their PA levels at school (Mayorga-Vega et al., 2018). Also, it provides adolescents with health literacy skills that empower them to be physically active outside school (Slingerland and Borghouts, 2011).

Although one of the main PE national standards worldwide is the promotion of studentsʼ lifelong PA (European Commission/EACEA/Eurydice, 2013; SHAPE America, 2013), there are several limitations that make difficult its achievement (Casado-Robles et al., 2019). For example, the large volume of curricular objectives and contents that have to be developed in a fairly limited time (Casado-Robles et al., 2019) or the difficulty of controlling studentsʼ PA outside school (Casado-Robles et al., 2019). A feasible solution could be promoting PA practice through the application of the intermittent teaching unit structure proposed by Viciana and Mayorga-Vega (2016). Intermittent teaching units are defined as those teaching units given in small temporary periods during all the lessons of the course or of a specific period (Viciana and Mayorga-Vega, 2016). For instance, this innovative structure of the teaching unit could consist of only a few minutes of each PE lesson (e.g., the last 15 min) for several lessons. Thus, it allows for dividing the lesson into several parts (i.e., two) and developing two or more related curricular objectives during the same lesson. This could provide teachers a more efficient use of the limited PE time developing various goals during the lessons, and students the opportunity to establish relationships between different PE-related contents. Previous empirical studies have shown the effectiveness of this teaching unit structure in developing and maintaining different health-related physical fitness curricular objectives over time, such as cardiorespiratory fitness or strength (Mayorga-Vega et al., 2016; Guijarro-Romero et al., 2020). Nevertheless, to date it has not been applied for promoting studentsʼ PA practice.

Additionally, even though PE is potentially considered a key context to promote studentsʼ PA (Association for Physical Education, 2020; World Health Organization, 2020), most PE-based interventions seem to show small or non-significant effects on increasing students’ PA levels (Borde et al., 2017; Love et al., 2019). Furthermore, Nguyen et al. (2016) observed that intervention effects diminish over the time, which may compromise long-term effectiveness of PA promotion interventions. The lack of theoretical behavior change frameworks in the design of school-based interventions could be the cause of these small effect sizes (Borde et al., 2017; Rhodes et al., 2017). Therefore, applying PE-based PA interventions, specifically grounded in theoretical frameworks of behavior change, could help to enhance motivational outcomes in PE and leisure-time PA context, as well as to maintain the beneficial effects of an intervention over time (Kwasnicka et al., 2016; Rhodes et al., 2017).

The Self-Determination Theory (SDT) is a motivational theory widely used to understand the antecedents and consequences of studentsʼ motivation toward PA both in the school context of PE and in the out-of-school context (Deci and Ryan, 1985; Ryan and Deci, 2020). The SDT considers motivation as a multidimensional construct with different levels along a *continuum* according to the degree of autonomy, ranging from more self-determined (i.e., autonomous) to less self-determined (i.e., controlled) forms of behavioral regulations (Ryan and Deci, 2020). Autonomous forms of motivation include intrinsic motivation, as well as integrated, and identified regulation (Ryan et al., 2021). Intrinsic motivation is related to the inherent pleasure and satisfaction provided by an activity; integrated regulation refers when activity is completely assimilated with individual’s sense of self; and identified regulation refers when an activity is aligned with personal values (Ryan and Deci, 2020). Controlled forms of motivation comprise introjected (i.e., acting to avoid sense of guilt or anxiety or to protect contingent self-worth) and external (i.e., doing an activity for contingent rewards or avoid punishments) regulations (Ryan and Deci, 2020; Ryan et al., 2021). Finally, amotivation is characterized by no intention of an individual to act due to different reasons such as lack of certain skills or knowledge necessary to act (Ryan and Deci, 2020). The SDT postulates that everyone has three basic psychological needs: autonomy, which refers to the need for initiative and ownership in one’s own behavior; competence, which refers to the need to feel capable of performing a behavior effectively, and relatedness, which refers to the need of belonging and connection by significant others (Ryan and Deci, 2020). Satisfaction of these three basic psychological needs, leads students to acquire more autonomous forms of motivation toward PA (Ryan and Deci, 2020). Furthermore, according to the SDT the autonomy support of PE teachers in PE plays a key role in the development of a more autonomous motivation toward PA (Ryan and Deci, 2020), which is positively associated with the interest in remaining active in the out-of-school time (Sevil-Serrano et al., 2022). The autonomy-supportive teaching style is characterized by making students feel that they can participate in their own learning (Ryan and Deci, 2020). In this sense, Stefanou et al. (2004) proposed that autonomy support could be characterized by three clearly different dimensions: organizational (i.e., autonomy in terms of encouraging students’ ownership of decisions in relation to PA), procedural (i.e., autonomy in terms of encouraging studentsʼ to conduct the teaching-learning process), and cognitive (i.e,, autonomy in terms of promoting studentsʼ ownership to express and argue their particular viewpoint in the teaching-learning process). According to SDT framework, the application of an autonomy-supportive teaching style and the improvement of students’ autonomous motivation are considered key determining factors related to the acquisition and maintenance of students’ PA (Teixeira et al., 2012).

Based on the SDT and on the Theory of Planned Behavior (Ajzen, 1991), the trans-contextual model (TCM) was developed to conceptualize the transfer of self-determined motivation from educational contexts to out-of school contexts (Hagger and Chatzisarantis, 2016; Viciana et al., 2019). The premise behind this model is that motivation in these two contexts are likely to be highly salient in determining studentsʼ intentions and participation in PA. The TCM proposes three empirically testable propositions to explain the mechanisms by which teachersʼ support for motivation in school PE affects studentsʼ involvement in PA (Hagger et al., 2003). Firstly, studentsʼ perceptions of PE teachersʼ autonomy support are proposed to be related to their autonomous motivation toward activities performed in PE. Secondly, autonomous motivation in PE will predict autonomous motivation toward similar activities in leisure-time contexts. Thirdly, autonomous motivation in leisure-time contexts will predict studentsʼ future intentions to participate in similar activities, as well as actual behavioral engagement (Hagger and Chatzisarantis, 2016; Viciana et al., 2019). Specifically, the model suggests that attitude (i.e., studentsʼ perception of negative or positive evaluation of carrying out PA), subjective norm (i.e., studentsʼ perception of normative expectations to carry out or not to carry out PA), and perceived behavioral control (i.e., studentsʼ beliefs of ease or difficulty to perform PA) seem to be positively influenced by students’ autonomous motivation for leisure-time PA (González-Cutre et al., 2014a,b). As a consequence, attitude, subjective norm, and perceived behavioral control will positively influence PA intention, which will ultimately manifest in behaviors that will encourage students to integrate PA as part of their lives (González-Cutre et al., 2014a,b; Hagger and Chatzisarantis, 2016). The systematic review conducted by Hagger and Chatzisarantis (2016) showed empirical support of the TCM across multiple studies conducted in the PE context, highlighting significant relationships between perceived autonomy support and self-determined motivation in PE (i.e., first proposition), between self-determined motivation in PE and in PA (i.e., second proposition), and between self-determined motivation and intention toward PA and actual PA engagement (i.e., third proposition).

In addition to the aforementioned individual theories related to studentsʼ PA promotion, a recent systematic review and meta-analysis found that the inclusion of behavior change techniques (Abraham and Michie, 2008) such as PA self-monitoring (e.g., activity wristbands) as part of the PA promotion programs has shown to be an effective strategy in increasing studentsʼ motivation toward PA practice, as well as the practice itself (Casado-Robles et al., 2022a,b). Furthermore, this systematic review and meta-analysis also found that incorporating a greater number of behavior modification strategies (e.g., educational counseling lessons or goal settings; Abraham and Michie, 2008) have also shown to be effective for promoting motivation toward PA among students from the PE setting (Casado-Robles et al., 2022a,b). In this sense, only four studies in the PE setting have examined the effects of intervention programs based on the use of activity wristbands, either alone or in combination with other behavior modification strategies, on high school students’ PA practice promotion (Casado-Robles et al., 2022a,b). Unfortunately, to our knowledge, no previous studies have examined the effect of intermittent teaching units based on the use of activity wristbands and behavior modification strategies on studentsʼ PA practice mediators and PA practice itself. Additionally, although previous research has evidenced TCM to be a useful framework for understanding the role of self-determined motivation in PE in fostering PA (Hagger and Chatzisarantis, 2016), the current research evidence of PE-based intervention studies according to the postulates of the TCM is still scarce (Casado-Robles et al., 2022a). Especially incorporating innovative teaching unit structures together with activity wristbands and behavior modification strategies. The present study was designed to address these needs from a holistic perspective by investigating the effectiveness of an intermittent teaching unit based on the use of activity wristbands and behavior modification strategies and promoting an autonomy-supportive teaching style. Consequently, the main objective of the present study was to examine the effects of an intermittent teaching unit based on the use of activity wristbands and behavior modification strategies on high school studentsʼ perceptions of autonomy support, satisfaction of basic psychological needs, autonomous and controlled motivation toward PE and PA, intention to be physically active, and habitual PA levels. In line with the TCM tenets (Hagger and Chatzisarantis, 2016) and previous research that provide empirical support of the application of the TCM in the PE context (Sevil-Serrano et al., 2022), the main hypotheses were that students who perform the intermittent teaching unit based on the use of activity wristbands and behavior modification strategies will manifest: a) higher perceptions of autonomy support, satisfaction of basic psychological needs, and in consequence, higher autonomous and controlled motivation toward PE; b) higher motivation toward PA, intention to be physically active, and as a consequence, higher habitual PA levels compared with students in the control condition.

## 2. Materials and methods

### 2.1. Study design

The present study is reported according to the CONSORT for cluster-randomized trials guidelines (Campbell et al., 2012). The protocol conforms to the Declaration of Helsinki statements (64th WMA, Brazil, October 2013) and was approved by the Ethical Committee for Human Studies at the University of Granada (code number: 1252/CEIH/2020). Recruitment of participants was carried out in December of 2021, and the intervention was done from January 2022 to May 2022. For practical reasons and due to the nature of the present study (i.e., pre-established classes in a school setting), a cluster randomized controlled trial design was used (Casado-Robles et al., 2022a). This study was non-blinded (treatments were not masked from the students or teacher), and parallel-grouped (study with two different treatments; Spieth et al., 2016), with three evaluation phases. The study has been registered as a clinical trial (ID: NCT05949463).

### 2.2. Participants

Firstly, the principal and the PE teachers of two state high-school centers of the province of Granada (Granada, Spain) chosen by convenience were contacted and informed about the study, requesting permission to conduct it. After obtaining the approval to carry out the present study, all 353 students (47.5% females) from the seventh to tenth grades of secondary education (i.e., 12–16 years old) were invited to participate in it. Students and their legal tutors were fully informed about the study features. Participants’ signed written informed assent and their legal tutors’ signed written informed consent were obtained before taking part in the study. According to the center’s reports, all the students’ families had a middle socioeconomic level.

The inclusion criteria were: a) being enrolled in the seventh to tenth grade at the secondary education level; b) participating in the normal PE lessons; c) being exempt of any health problem that would make them unable to engage in PA normally; d) presenting the corresponding signed written consent by their legal tutors, and e) presenting their own corresponding signed written assent. The exclusion criterion was defined as not having performed the evaluation of the dependent variables correctly at the pre-intervention, post-intervention and/or follow-up measures following the administration rules (being removed only for incomplete variables and not for the overall study).

### 2.3. Sample size

*A priori* sample size calculation was estimated with the Optimal Design Plus Empirical Evidence Software Version 3.01 for Windows. Parameters were set as follows: significance level α = 0.05, number of participants per cluster *J* = 16, effect size *δ* = 0.60 (Casado-Robles et al., 2022b), intra-class correlation coefficient *ρ* = 0.01, statistical power (1 – β) = 0.80, and dropout = 20% (Howie and Straker, 2016). A minimum final sample size of at least 112 participants (minimum initial sample size equal to 135) was estimated.

### 2.4. Randomization

To avoid contamination of treatments, randomization was conducted at the school-level, using a computerized random number generator. This was done before the pre-intervention evaluation was administered by an independent researcher blinded to the study aim and following a 1:1 ratio into the intermittent (IG) or control (CG) groups.

### 2.5. Measures

Data collection was carried out at the beginning and at the end of the teaching unit (pre-intervention and post-intervention, respectively), as well as at the end of the follow-up period. All evaluations were performed during two PE lessons’ time by the same tester, instruments, and protocols. Prior to carrying out the intervention, students’ sex and age information were obtained from school reports. Additionally, the students’ anthropometric measures were taken following the International Standards for Anthropometric Assessment (Stewart et al., 2011). Moreover, students’ previous experience with activity wristbands and current use of them were collected. Then, the first half of two PE lessons were used to evaluate students’ psychological perceptions (i.e., autonomy support, basic psychological needs, motivation, and intention to be physically active) and self-reported habitual PA under silent conditions, leaving the second half for PA practice. At the beginning of the evaluation sessions, the researcher provided a complete explanation about how to correctly fill out the questionnaire. Students were asked for their maximum sincerity, and they were guaranteed the confidentiality of the obtained data. Although instructions on how to correctly respond to the questionnaire were printed at the top, the researcher was present during the whole evaluation session to clarify any question that might arise.

#### 2.5.1. Anthropometric

Participants’ body mass and height were measured in shorts, T-shirts, and barefoot. For the body mass measure, the student stood in the center of the scale (Seca, Ltd., Hamburg, Germany; accuracy = 0.1 kg) without support and with their weight distributed evenly on both feet. For the body height assessment, participants stood with their feet together with the heels, buttocks, and upper part of the back touching the stadiometer (Holtain Ltd., Crymmych, Pembs, United Kingdom; accuracy = 0.1 cm), and with the head placed in the Frankfort plane. Two measurements of both body mass and height were performed and the average of each was calculated (Stewart et al., 2011). Then, the body mass index was calculated as body mass divided by body height squared (kg/m^2^). Finally, students’ body weight status was categorized by the body mass index cut-points as overweight/obese (i.e., sex- and age-adjusted cut-point values equal to or higher than the equivalent value of 25 kg/m^2^ at the age of 18 years) or non-overweight/obese (i.e., lower than the above-mentioned cut-point values) (Cole et al., 2000).

#### 2.5.2. Perceived autonomy support

The PE teacher autonomy-support was assessed through the Spanish version of the Multi-Dimensional Perceived Autonomy Support Scale for PA (MD-PASS-PA; Trigueros et al., 2020). It consists of 15 items (five items per factor) that assessed organizational (e.g., “My PE teacher allows me to choose different exercise or sports options in my free time”), procedural (e.g., “My PE teacher gives me an overview of the different types of physical or sports exercise I can do in my free time”), and cognitive (e.g., “My PE teacher allows me to express my opinion about the physical exercise or sport I do in my free time”) autonomy support. The Spanish version of MD-PASS-PA has shown adequate psychometric properties among adolescents (CFI = 0.98; IFI = 0.92; TLI = 0.98; SRMR = 0.04; RMSEA = 0.05; Cronbach’s α = 0.82–0.86) (Trigueros et al., 2020).

#### 2.5.3. Basic psychological needs

Students’ perceptions of autonomy, competence, and relatedness satisfaction in PE and physical exercise were assessed using the Spanish version of the Basic Psychological Needs in Exercise Scale (BPNES; Sánchez and Núñez, 2007). It consists of 12 items (four items per factor) that assessed autonomy (e.g., “The physical exercise program that I follow is closely related to what I like and what interests me”), competence (e.g., “I think I have made tremendous progress with respect to the final goal that I pursue”), and relatedness (e.g., “I feel very comfortable with the other participants in the physical exercise program”). The items were preceded by the statement “When I do PA….” The Spanish version of BPNES has shown adequate psychometric properties among adolescents (CFI = 0.95; IFI = 0.95; SRMR = 0.05; RMSEA = 0.08; Cronbach’s α = 0.74–0.87) (Sánchez and Núñez, 2007).

#### 2.5.4. Motivation toward physical education

Participants’ self-determined motivation toward PE was measured by the Spanish version of the Revised Perceived Locus of Causality Scale (PLOC-R; Trigueros et al., 2017). It consists of of 23 items spread over six dimensions (four items each except external regulation) that measure intrinsic motivation (e.g., “Because PE is nice”), amotivation (e.g., “But I do not really know why”), integrated (e.g., “Because it agrees with my way of life”), identified (e.g., “Because it is important for me to do PE”), introjected (e.g., “Because I would feel bad about myself if I did not”), and external regulation (e.g., “Because it is compulsory”). This questionnaire was preceded by the statement “I participate in PE lessons….” The autonomous (i.e., averaging intrinsic, integrated, and identified regulation) and controlled (i.e., averaging introjected and external) motivations were also calculated (Chemolli and Gagné, 2014). The Spanish version of the PLOC-R has shown adequate psychometric properties among adolescents (CFI = 0.95; TLI = 0.94; IFI = 0.95; SMR = 0.038 RMSEA = 0.067; Cronbach’s alpha = 0.86–0.92) (Trigueros et al., 2017).

#### 2.5.5. Self-determined motivation toward physical activity

Students’ motivation toward PA was measured using the Spanish version of the Behavioral Regulation in Exercise Questionnaire (BREQ-3; González-Cutre et al., 2010). It consists of 23 items distributed into six dimensions (four items each except identified regulation) that measure intrinsic motivation (e.g., “Because I think exercise is fun”), amotivation (e.g., “I think exercising is a waste of time”), integrated (e.g., “Because it agrees with my way of life”), identified (e.g., “Because it is important to me to exercise regularly”), introjected (e.g., “Because I feel guilty when I do not practice it”), and external (e.g., “Because the others tell me that I should do it”) regulation. This questionnaire was preceded by the statement: “I do PA…” The autonomous and controlled motivations were also calculated (Chemolli and Gagné, 2014). The Spanish version of the BREQ-3 has shown adequate psychometric properties among high-school students (CFI = 0.91; IFI = 0.91; RMSEA = 0.06; SRMR = 0.06; Cronbach’s α = 0.66–0.87) (González-Cutre et al., 2010).

#### 2.5.6. Intention to be physically active

Students’ intention to be physically active in their free time was measured using the Spanish version of the Intention to partake in leisure-time PA questionnaire (Granero-Gallegos et al., 2014). It is composed of three items (e.g., “I intend to exercise at least three times a week for the next month”). The items were preceded by the sentence: “In my free time, after school…” The Spanish version of this questionnaire has shown adequate psychometric properties among adolescents (GFI = 1.00; RMR = 0.02; NFI = 1.00; NNFI = 0.99; CFI = 1.00; RMSEA = 0.03; Cronbach’s α = 0.93) (Granero-Gallegos et al., 2014).

To adapt the scale of the five previous questionnaires to the Spanish students’ school grades, a 10-point Likert-type was used according to previous studies (Casado-Robles et al., 2022a).

#### 2.5.7. Habitual physical activity

Students’ habitual PA was measured using the Physician-based Assessment and Counseling for Exercise questionnaire (PACE; Martínez-Gómez et al., 2009). It consists of two questions that measure how many days in the last week and in a habitual week at least 60 min of PA are performed. A 7-point Likert-type scale, ranging from 0 to 7 was used. The PACE questionnaire has shown adequate psychometric properties among high-school students (r = 0.43) (Martínez-Gómez et al., 2009).

### 2.6. Intervention

Both IG and CG students were required to participate in two mandatory PE lessons per week during the intervention period. Before the intervention, grounded in the SDT the PE teacher was trained to be autonomy and need-supportive during the intervention. The PE teacher autonomy and need-support training was focused on motivation and behavior change techniques provided by Teixeira et al. (2020). Furthermore, the guidelines for correctly delivering the lessons of the IG were designed by the researchers and given to the PE teacher. The main researcher supervised all the lessons and made sure all guidelines were taken into account during the program.

The IG students performed an intermittent teaching unit (Viciana and Mayorga-Vega, 2016) twice a week for eight weeks aimed at promoting healthy PA habits. Specifically, the last 15 min of each lesson were used. The rest of lessons’ time other contents were worked on with no relation to any health PA habit (i.e., acrosport, badminton, basketball, volleyball, soccer and athletics). Based on recent literature (Casado-Robles et al., 2022b) as the main strategy of the teaching unit to promote regular PA practice, students wore an activity wristband the whole day (Xiaomi Mi Band 5) during the development of it. Reminders for an inactivity alert, achieved goal alert, and event alert for school recess and weekends were activated. In addition to the steps number, the activity wristband also measures heart rate, blood pressure, sleep time, distance, and calories. However, these options were hidden for participants in the activity wristband. At the beginning of the intervention, the main objective and activity wristband operation, including their specific mobile application (Mi Fit), were explained. Furthermore, with the aim of increasing participants interest, motivation, and commitment both teacher and IG participants signed a behavior change contract named “I improve my PA level” at the beginning of the intervention. Together with the contract, participants received a graph in which they had to reflect the average number of steps taken each week (data obtained from the Xiaomi Mi Fit). Next, participants of each class were organized in groups of four. Based on their preintervention PA levels, from the second to the last week of the intervention, a competition that consisted of achieving progressive step challenges, both individually and in teams, was established. During these last 15 min of the lessons, the information recorded by the activity wristband was analyzed in order to provide students with feedback of their daily PA habits. Additionally, together with the use of activity wristbands and PA goal challenges, educational counseling was applied (Casado-Robles et al., 2022b) in order to be need-supportive. Grounded in SDT motivation and behavior change techniques proposed by Teixeira et al. (2020), educational counseling included information about: (1) the benefits of regular PA practice; (2) PA recommendations, (3) types of PA that can be performed depending on the intensity and sedentary activities to avoid (PA pyramid), (4) healthy PA practice proposals for periods such as recess and leisure time, (5) barriers toward PA practice and their possible solutions, (6) sensitizing videos and news about PA practice, and (7) a list of 10 healthy behaviors. Also as part of the intervention, a personalized blog^1^ with all the resources created for the intervention (e.g., tutorials of the activity wristband and mobile application operation, posters of the PA pyramid, PA recommendations, a list of 10 healthy behaviors, a list of PA practice proposals, barriers toward PA and solutions, as well as audiovisual material) was created. Moreover, a WhatsApp group was created for the IG in order to: a) promote students’ satisfaction of basic psychological needs sending, every week from Monday to Saturday, six text messages (reviewed by a SDT specialist) (Ludwig et al., 2018), reminding students to synchronize the activity wristband with the mobile application (on Sunday), as well as to change the PA steps challenge. Finally, after the intervention, participants completed a six-week period in which they were encourage to autonomously maintain the PA challenge of 10,000 steps per day. They continued wearing the activity wristbands with the same reminders activated and were also encouraged to continue synchronizing the data of the activity wristband with the mobile application every week. However, no additional behavior modification strategies were applied.

Regarding the CG students, they also carried out two PE lessons a week during the intervention period. During these lessons, contents of handball, basketball, alternative sports and traditional games were developed. However, this group did not wear activity wristbands or receive any behavior modification specific strategy developed in the IG.

### 2.7. Statistical analysis

Descriptive statistics (mean ± standard deviation or percentage) for the general characteristics of the participants and dependent variables were calculated. Firstly, all the statistical tests assumptions were first checked for each dependent variable by common procedures (e.g., histograms and normal Q-Q plots for normality). Then, as exploratory analyses, the one-way analyses of variance (ANOVA) (continuous variables) and the chi-squared test (categorical variables) were conducted to examine potential differences in terms of general characteristics between the two groups. The internal consistency of the dependent variables measured by the questionnaires was examined with the Cronbach’s alpha. Afterward, the effect of the activity wristband-based teaching unit on students’ questionnaires scores was examined. All the participants were included in the statistical analyses regardless of adherence to the protocol (i.e., intention-to-treat approach). All the participants that did not follow protocol had failed to sustain a 100% attendance rate. Because the unit of intervention was the class, a Multilevel Linear Model with participants nested within classes and measures nested within participants as random effects, and with the between-group factor *group* (control, intermittent) and the within-group factor *time* (pre-intervention, post-intervention, follow-up) as fixed effects was selected (i.e., two-way mixed nested ANOVA/ANCOVA; Li et al., 2017). All the potential confounding variables (i.e., sex, grade, body mass, body height, body mass index, previous and current use of activity wristbands, and intervention attendance) were explored and used as covariables when necessary (see Table 1 footnotes). The maximum likelihood estimation method was used. Then, the *post-hoc* within-group pairwise comparisons with the Bonferroni adjustment for each group independently was carried out. Effect sizes were estimated using the Cohen’s *d*. Finally, although an intention-to-treat approach was followed in the present study, as a sensitivity analyses, all the above-mentioned analyses were also carried out with a per-protocol approach (i.e., including the participants, taking into consideration their adherence to the protocol, that is, 100%). All statistical analyses were performed using the SPSS version 25.0 for Windows (IBM® SPSS® Statistics). The statistical significance level was set at *p* ≤ 0.05.

## 3. Results

### 3.1. Final sample and general characteristics

**Figure 1:**
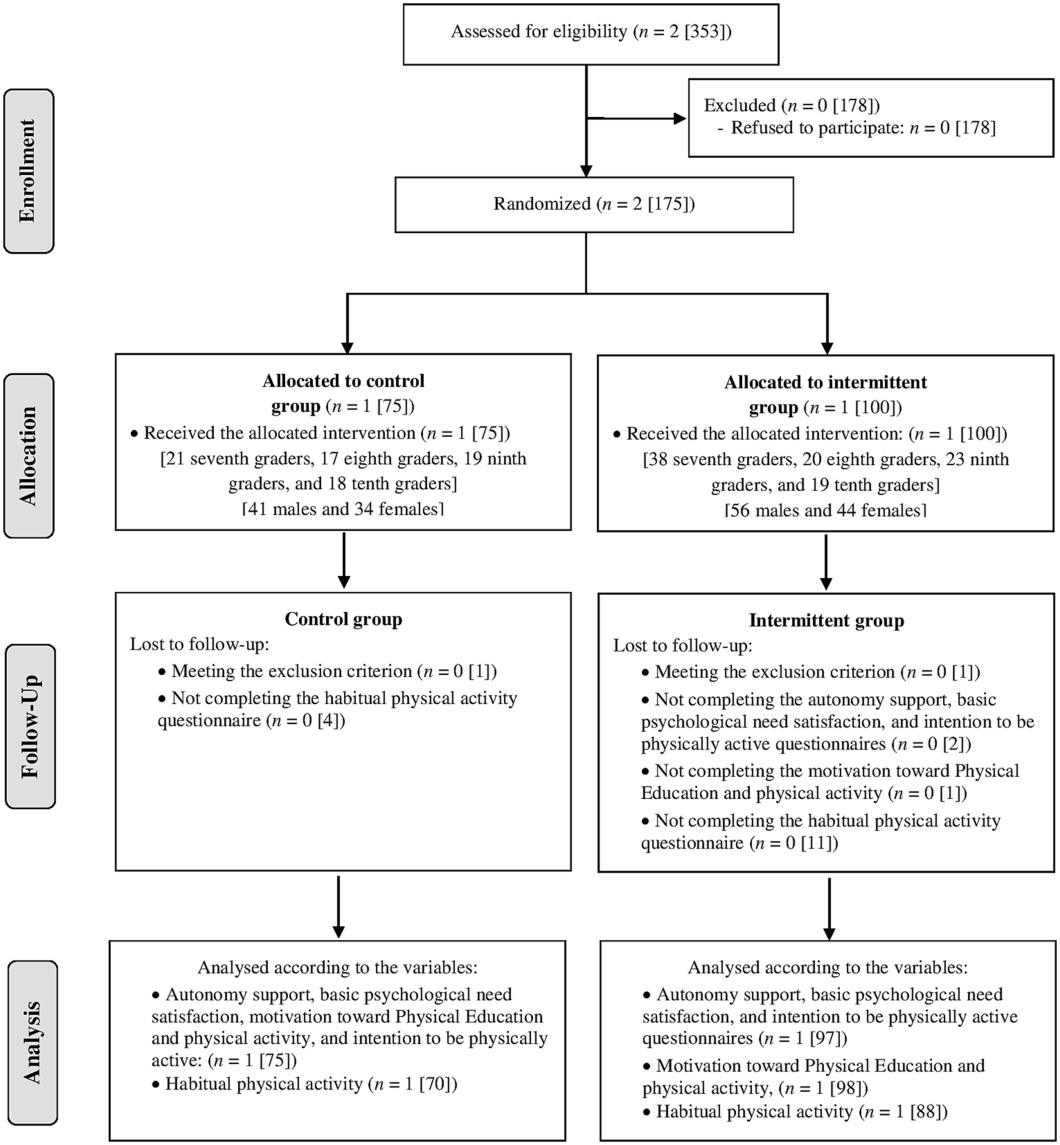
Flow chart of participants included in the present study. All numbers are school centers [students].

Figure 1 shows the flow chart of the participants included in the present study. From the 353 students that were invited to participate in the present study, 175 students (45.1% females) agreed and met the inclusion criteria. However, 173 students (45.7% females) passed the exclusion criterion. A total of 70–75 and 88–98 participants from CG and IG, respectively, had dependent variables with completed data in the three measures. Table 2 shows the general characteristics of the included participants. The results of the one-way ANOVA and the chi-square test did not show statistically significant differences in terms of general characteristics between the two groups (*p* > 0.05), except for the body height and weight status (*p* < 0.05). Regarding the attendance rate, the IG participants obtained an average of 92.6% (number of participants with an attendance rate 50–59% = 3; 60–69% = 3; 70–79% = 4; 80–89% = 22; 90–99% = 8; 100% = 58). In the sample of the present study, the internal consistency of all the dependent variables measured by dimensional questionnaires was above 0.70 (from 0.76 to 0.92).

**Table 2:** General characteristics of the included participants.

	Total (*n* = 173)	Control (*n* = 75)	Intermittent (*n* = 98)	*F/* χ*^2^*	*p^a^*
Age (years)^b^	13.3 (1.2)	13.4 (1.2)	13.2 (1.3)	1.045	0.308
Sex (females/males)^c^	45.7/54.3	44.0/56.0	46.9/53.1	0.148	0.701
Grade (7^th^/8^th^/9^th^/10^th^)^c^	34.1/21.4/24.3/20.2	28.0/22.7/25.3/24.0	38.8/20.4/23.5/17.3	2.538	0.468
Body mass (kg)^b^	55.2 (12.3)	56.1 (10.3)	54.5 (13.7)	0.721	0.397
Body height (cm)^b^	161.6 (9.5)	164.0 (9.6)	159.8 (9.1)	8.759	0.004
Body mass index (kg/m^2^)^b^	21.0 (3.8)	20.8 (2.9)	21.2 (4.3)	0.553	0.458
Overweight-obese (no/yes)^c^	74.4/25.6	82.7/17.3	68.0/32.0	4.752	0.029
Previous experience with activity wristbands (no/yes)^c^	29.7/70.3	31.1/68.9	28.6/71.4	0.127	0.721
Current use of activity wristbands (no/yes)^c^	69.8/30.2	63.5/36.5	74.5/25.5	2.408	0.121
Habitual PA (days/week)^b,d^	3.6 (1.6)	3.5 (1.7)	3.7 (1.6)	0.355	0.552

^a^Significance level from the one-way analysis of variance for continous variables and the chi squared test for categorical variables; Data are reported as mean (standard deviation) for continous variables^b^ and as percentage for categorical variables^c^; ^d^Baseline (pre-intervention) PACE scores.

### 3.2. Effect of the activity wristband-based teaching unit on students’ questionnaires scores

Table 1 shows the effect of the intermittent teaching unit on students’ questionnaires scores. The Multilevel Linear Model results, followed by the within-group pairwise comparisons, showed that the IG students statistically significantly improved cognitive and procedural autonomy scores from pre- to post-intervention (*p* < 0.05), as well as procedural autonomy scores from pre-intervention to follow-up (*p* < 0.01); and they also statistically significantly improved autonomy and relatedness basic psychological needs, and autonomous motivation toward PA scores from post-intervention to follow-up, and from pre-intervention to follow-up (*p* < 0.05).

**Table 1:** Effect of the intermittent teaching unit on students’ questionnaires scores.

	Pre-intervention (1)	Post-intervencion (2)	Follow-up (3)	Multilevel linear model^a^			Effect sizes (*d*)^b^		
	Mean (SE)	Mean (SE)	Mean (SE)	*- 2LL*	*F*	*p*	1–2	2–3	1–3
*Cognitive autonomy*									
Control (*n* = 75)	7.3 (0.2)	7.1 (0.2)	7.1 (0.2)	1863.012	3.182	0.044	0.34	−0.05	0.28
Intermittent (*n* = 97)	8.7 (0.2)	9.1 (0.1)*	9.0 (0.2)						
*Procedural autonomy*									
Control (*n* = 75)	6.4 (0.2)	6.3 (0.2)	6.4 (0.2)	2005.722	8.956	< 0.001	0.61	−0.06	0.55
Intermittent (*n* = 97)	7.7 (0.2)	8.8 (0.2)***	8.8 (0.2)‡‡‡						
*Organizational autonomy*									
Control (*n* = 75)	6.9 (0.2)	7.1 (0.2)	7.0 (0.2)	1885.318	1.379	0.254	0.20	0.04	0.24
Intermittent (*n* = 97)	8.4 (0.2)	9.0 (0.2)**	8.9 (0.2)‡‡						
*BPN autonomy*									
Control (*n* = 75)	7.8 (0.2)	7.6 (0.2)	7.7 (0.2)	1944.602	5.082	0.007	0.10	0.35	0.45
Intermittent (*n* = 97)	7.9 (0.2)	8.0 (0.2)	8.7 (0.2)†††/‡‡‡						
*BPN competence*									
Control (*n* = 75)	7.8 (0.2)	7.7 (0.2)	7.9 (0.2)	2016.680	0.343	0.710	0.02	0.10	0.12
Intermittent (*n* = 97)	8.5 (0.2)	8.5 (0.2)	8.8 (0.2)						
*BPN relatedness^c^*									
Control (*n* = 75)	7.7 (0.2)	7.7 (0.2)	7.8 (0.2)	1875.821	5.943	0.003	0.09	0.34	0.43
Intermittent (*n* = 97)	7.9 (0.2)	8.0 (0.2)	8.7 (0.2)†††/‡‡‡						
*Autonomous motivation toward PE^d^*									
Control (*n* = 75)	6.8 (0.2)	6.9 (0.2)	7.2 (0.2)	2038.821	0.108	0.898	0.05	0.01	0.07
Intermittent (*n* = 98)	7.8 (0.2)	8.0 (0.2)	8.3 (0.2)						
*Controlled motivation toward PE*									
Control (*n* = 75)	4.7 (0.3)	5.0 (0.2)	4.9 (0.3)	2254.403	2.155	0.118	−0.02	0.35	0.33
Intermittent (*n* = 98)	4.7 (0.2)	5.0 (0.2)	5.6 (0.2)†/‡‡						
*Autonomous motivation toward PA^d^*									
Control (*n* = 75)	7.4 (0.2)	7.6 (0.2)	7.4 (0.2)	1954.631	4.402	0.013	−0.12	0.39	0.27
Intermittent (*n* = 95)	7.7 (0.2)	7.7 (0.2)	8.2 (0.2)††/‡						
*Controlled motivation toward PA*									
Control (*n* = 75)	3.2 (0.2)	4.2 (0.2)***	4.2 (0.2)‡‡‡	2061.107	10.682	< 0.001	−0.51	−0.26	−0.77
Intermittent (*n* = 96)	3.8 (0.2)	3.8 (0.2)	3.4 (0.2)						
*Intention to be physically active*									
Control (*n* = 75)	8.4 (0.3)	8.1 (0.3)	8.5 (0.2)	2152.298	0.651	0.522	0.15	−0.16	−0.01
Intermittent (*n* = 97)	8.7 (0.2)	8.8 (0.2)	8.8 (0.2)						
*Habitual PA^c^*									
Control (*n* = 75)	3.5 (0.2)	3.8 (0.2)	3.7 (0.2)	1781.809	5.308	0.006	0.10	0.33	0.43
Intermittent (*n* = 98)	3.7 (0.2)	4.2 (0.2)**	4.6 (0.2)†/‡‡‡						

SE, Standard error; - 2LL = -2 log-likelihood; BPN, Basic psychological needs; PE, Physical Education; PA, Physical activity; ^a^Multilevel Linear Model with participants nested within classes and measures nested within participants as random effects, and with the between-subjects factor group (control, intermittent) and the within-subject factor time (pre-intervention, post-intervention, follow-up) as fixed effects was selected (i.e., two-way mixed nested ANOVA/ANCOVA); *Post-hoc* within-subject pairwise comparisons with Bonferroni adjustment for each group independently: Pre-post-intervention change (* *p* < 0.05, ** *p* < 0.01, *** *p* < 0.001); Post-intervention-post-maintenance change († *p* < 0.05, †† *p* < 0.01, ††† *p* < 0.001), and pre-intervention-post-maintenance change (‡ *p* < 0.05, ‡‡ *p* < 0.01, ‡‡‡ *p* < 0.001); The covariables used for each analysis were as follows: ^c^Sex and ^d^Body height; ^b^Cohen’s d effect size.

Moreover, the results showed that IG students statistically improved the habitual PA scores from pre- to post-intervention, from post-intervention to follow-up, and from pre-intervention to follow-up (*p* < 0.05). However, the Multilevel Linear Model results, followed by the within-group pairwise comparisons, showed that the CG students statistically significantly improved controlled motivation toward PA scores from pre- to post-intervention and from pre-intervention to follow-up (*p* < 0.001). On the other hand, for the rest of variables statistically significant differences were not found (*p* > 0.05).

### 3.3. Sensitivity analysis

Supplementary Table S1 shows the sensitivity analysis (i.e., per-protocol approach) of the effect of the intermittent teaching unit on students’ questionnaires scores. The results with the per-protocol approach found similar outcomes as with the main analysis (i.e., intention-to-treat approach). Exceptionally, the Multilevel Linear Model results, followed by the within-group pairwise comparisons, showed that with the per-protocol approach the IG students statistically significantly improved controlled motivation toward PE scores from pre-intervention to follow-up (*p* < 0.001; for the main analyses the interaction effect *p* value was >0.05). Moreover, while with both approaches IG students statistically significantly improved autonomous motivation toward PA scores from post-intervention to follow-up (*p* < 0.01), with the per-protocol approach was not statistically significant from pre-intervention to follow-up (*p* > 0.05) as it was for the intention-to-treat approach (*p* < 0.05).

## 4. Discussion

The main objective of the present study was to examine the effects of an intermittent teaching unit based on the use of activity wristbands and behavior modification strategies on high school studentsʼ perceptions of autonomy support, satisfaction of basic psychological needs, autonomous and controlled motivation toward PE and PA, intention to be physically active, and habitual PA levels. Little research has examined the effects of intervention programs based on the use of activity wristbands on adolescents PA practice mediators, either alone or in combination with other strategies following the tenets of TCM. Results of this study have shown that the intermittent teaching unit improved studentsʼ cognitive and procedural autonomy support, as well as habitual PA levels from pre- to post-intervention. Interpreting the results under the tenets of the TCM, it seems that the PE intervention, centered on the promotion of studentsʼ autonomy for the participation in PA could increase their perception of autonomy support. This could be due to that: 1) the intervention was based on a continuous analysis of the information recorded by the activity wristband which provided students with feedback of their daily PA habits (Casado-Robles et al., 2022b), and 2) the educational PA counseling that included a wide variety of information about PA practice (Neil-Sztramko et al., 2021; Casado-Robles et al., 2022b). These findings are in line with previous school-based PA interventions where PE teachers support students’ leisure-time PA (González-Cutre et al., 2014a; Casado-Robles et al., 2022a; Sevil-Serrano et al., 2022). Regarding the absence of differences on the organizational autonomy factor, it could be because, although the PE teacher suggested a variety of activities that students could perform in different contexts (i.e., school recess and leisure time), most of them were based on achieving steps. Therefore, other activities like swimming, riding a bike or skipping rope, among others, were not suggested. This could lead students to perceived that these activities are the only ones that could be performed in the aforementioned contexts. However, it is important to highlight that they were suggestions, but students had the opportunity to choose what they considered more suitable to achieve the step goals proposed.

According to the TCM, autonomy support in PE will predict autonomous motivation in PE, which in turn influences his/her autonomous motivation toward similar activities in leisure-time contexts (Hagger and Chatzisarantis, 2016). Contrary to the TCM proposition (Hagger and Chatzisarantis, 2016) and past evidence (Cheon and Reeve, 2013; Sevil-Serrano et al., 2022), in the present study the increase showed in autonomy support was not translated into a positive effect in students’ motivational outcomes to leisure-time PA (i.e., basic psychological needs satisfaction and motivation toward PE and PA) from pre- to post-intervention. However, none of these studies used activity wristbands for promoting PA practice. Similar to the present study, Kerner and Goodyear (2017) examined the effect of wearing an activity wristband during eight weeks on studentsʼ motivation toward PA. These authors found that activity wristbands negatively impacted studentsʼ motivation toward PA. Nevertheless, this study did not apply any specific strategy during the intervention period. On the contrary, a recent systematic review and meta-analysis found that the inclusion of activity wristbands together with other behavior modification strategies as part of the PA promotion programs is an effective strategy to increase studentsʼ motivation toward PA practice (Casado-Robles et al., 2022b). In this sense, although the teaching unit applied in this study was centered on teaching the students about the importance of practicing regular PA and included strategies that have shown to be effective for promoting studentsʼ PA practice mediators (e.g., motivation; Casado-Robles et al., 2022b), maybe the time used of each teaching unit lesson (i.e., 15 min), as well as the total length of the intervention (Neil-Sztramko et al., 2021), were not enough to produce an improvement in these variables. González-Cutre et al. (2016) pointed out that strategies of PA promotion require time to become operational and modify studentsʼ behavior. Moreover, group competition established during the intervention could also affect the improvement of studentsʼ autonomy, competence and relatedness needs, since when students lose in competitions or do not achieve the objective (i.e., progressive steps challenges), perceptions of competence and intrinsic motivation decrease (Kerner and Goodyear, 2017). Additionally, another possible explanation of the lack of change in these variables in the IG could be the high starting values of IG studens for these variables which would be difficult to increase (González-Cutre et al., 2014b). Perhaps, if the intervention included other support agents like parents or tutors (Sevil-Serrano et al., 2022), as well as an innovative methodology like gamification, which have been shown to be effective strategies for improving students’ motivation (Arufe-Giráldez et al., 2022), an increase in this variable could be achieved.

Regarding studentsʼ intention to be physically active, results of this study showed that the intermittent teaching unit did not influence this PA mediator. These results are opposite to previous studies carried out in the PE setting that have also applied autonomy-supportive teaching styles and have found that student’s intention to be physically active increased (Cheon and Reeve, 2013; Casado-Robles et al., 2022a). Nevertheless, results obtained in the present study with this variable seem logical considering that previous PA practice mediators according to the TCM (i.e., basic psychological needs and motivation) did not change either. Furthermore, another factor that could explain this result is the high values of intention to be physically active (8.7 out of 10 points) reported by IG students before the intervention. That is, it could be possible that students perceived that they already were motivated enough for PA practice without the need for extra motivation with the proposed intervention (Owen et al., 2014). Therefore, it is important to highlight that the maintenance of a high baseline motivation is an important achievement of the present study. In this sense, it seems necessary to study which specific strategies should be implemented in the interventions with these types of students to help them maintain their intention toward PA practice, or even to continue increasing it, therefore obtaining greater health-related benefits (World Health Organization, 2020). Surprisingly, after the intervention statistically significant differences were found in habitual PA levels of the IG students. These findings are very valuable, since although previous PA practice mediators did not increase, IG students reported that they performed PA with a higher frequency after the intervention. These results may be attributable to the higher perception in autonomy support from the PE teacher (González-Cutre et al., 2016), as well as by the use of PA wristbands and educational counseling provided during the intervention regarding the importance of practicing regular PA (Casado-Robles et al., 2022b). Additionally, although students’ motivational outcomes to leisure-time PA (i.e., basic psychological needs satisfaction, motivation toward PE and PA, and intention to practice) did not improve after the intervention, the high values maintained in all of them suggest that the third preposition of TCM could be confirmed (Hagger and Chatzisarantis, 2016), and are consistent with previous studies (González-Cutre et al., 2016; Casado-Robles et al., 2022a; Sevil-Serrano et al., 2022). Contrary to these results, Bronikowski et al. (2016) found that after an eight-week intervention using activity wristbands, self-reported PA assessed using the same self-report measure (Martínez-Gómez et al., 2009) did not change. A possible explanation could be that these authors only applied one strategy to modify studentsʼ PA behavior (i.e., use of activity wristband), which it has been suggested to be less effective in comparison with multi-dimensional interventions that combine several strategies to influence PA practice mediators (Casado-Robles et al., 2022a,b).

To verify whether the effects are maintained over time, a follow-up measurement 6 weeks after the end of the intervention was performed. The results revealed that habitual PA level changes were maintained and even improved, meanwhile differences in perceived autonomy support and intention to be physically active were not found. These results seem logical for the following reasons. Firstly, no autonomy support from PE was applied during this period of the study. Secondly, as it has been mentioned previously, strategies of PA promotion require time to become operational and modify studentsʼ behavior (González-Cutre et al., 2016). Consequently, these results suggest that it would be necessary to increase the length of interventions to achieve greater effectiveness in PA practice mediators. In this sense, although no behavior modification strategies were applied (except the use of an activity wristband), students had the objective of autonomously maintaining at least 10,000 steps per day. However, the main difference was that there was no competition between students for achieving more steps. This fact could lead students to feel more comfortable with this part of the intervention program, therefore contributing to experiencing higher perceptions of autonomy need, as well as better integration (i.e., relatedness need) with their peers. Regarding competence need, the establishment of a specific step target (i.e., 10,000 steps/day) to be autonomously maintained might be the cause of the absence of differences in this variable. According to previous literature (Kerner and Goodyear, 2017) when students are only focused on the PA outcome (step target) rather than the process, it could lead to feeling that they are unable to complete the step target set in the activity wristband, making them think they are failing, which could negatively impact their sense of competence.

Additionally, new positive differences were observed in autonomy and relatedness needs and autonomous motivation toward PA. On the basis of the theorical tenets of TCM (Hagger and Chatzisarantis, 2016), studentsʼ satisfaction of basic psychological needs in leisure-time PA could significantly contribute to autonomous motivation toward PE and this to autonomous motivation toward PA. Although motivation toward PE did not increase in a statistically significant manner from the post-intervention to the end of the follow-up period, it is important to highlight that the average punctuation in this variable was maintained and slightly increased, with already high values at the follow-up measurement. Finally, although no differences were found on studentsʼ intention to be physical, habitual PA levels increased again. In this sense, in addition to the responsibility of autonomously maintaining the PA practice with the help of activity wristbands that students had, the greater values in autonomous motivation toward PA obtained could help to increase their habitual PA levels (Hagger and Chatzisarantis, 2016). Previous similar studies carried out in the PE setting observed that the habitual PA levels decrease after the follow-up period (e.g., Ridgers et al., 2021). However, it is important to point out that (Ridgers et al., 2021) follow-up was six months after the end of the intervention. The later follow-up together with the fact that no additional objectives were proposed to students during this period of time (i.e., do your best steps per day) could be the reasons for the lack of differences in habitual PA levels.

The main strength of the present study was that, this is the first study that examines the effects of an intermittent teaching unit based on the use of activity wristbands and behavior modification strategies on studentsʼ perceptions of autonomy support, the satisfaction of basic psychological needs, autonomous and controlled motivation toward PE and PA, intention to be physically active, and habitual PA levels. Another strength is the inclusion of a follow-up measure to examine the long-term effectiveness of the program as suggested by previous literature (Nguyen et al., 2016). Moreover, because of the nature of the context (i.e., school) and with the objective of keeping the ecological validity, the use of a cluster-randomized controlled trial design (balanced by grade) was more appropriate for the present research objective (Campbell et al., 2012). Furthermore, the comparison with a CG that did not wear PA activity wristbands or receive any behavior modification strategy, allows us to check that the effects obtained are due to the intervention. Finally, the evaluation of the effect of the teaching unit with a Multilevel Linear Model with participants nested within classes and measures nested within participants as random effects, represents an advancement with respect to the commonly applied analyses (Li et al., 2017).

This study also has some limitations that should be acknowledged. Firstly, the non-probabilistic and relatively small sample size provides a lower generalization power. This limits the generalizability of the obtained outcomes to the particular studied population and context. However, due to human, time, and material resource restrictions, a probabilistic and larger sample could not be examined. Moreover, the teaching unit length could have been a limitation to achieving greater effects on the PA mediator variables. However, considering the large volume of objectives that have to be developed throughout the academic year with a very limited time for the PE subject (Casado-Robles et al., 2019), the purpose was to perform a real study that would be feasible to perform in the context of PE. Additionally, and according to the SDT framework which distinguishes six specific types of PE teacher interpersonal behaviors, only one of them was studied (i.e., autonomy support). Finally, SDT framework studied was only focused on the bright motivation path (i.e., need-supportive environment-need satisfaction-autonomous motivation-adaptive outcomes). That is, the need frustration and need-thwarting behaviors which are part of the dark motivational path were not studied. Future studies should include a probabilistic and larger sample, which provide a higher generalization of the obtained outcomes. Additionally, it would be interesting to reproduce the present study including additional social agents like parents or school teachers with the aim of checking if better results on studentsʼ PA practice mediators are obtained. Moreover, it would also be interesting reproducing this study and measuring PA objectively to check if the strategies applied really influence studentsʼ PA levels. Even, it would be interesting including a qualitative evaluation (i.e., interviews or focus groups) of the intervention program. This, would help us to deeply understand the specific perceptions and/or situations that students experience during the intervention. That is, the reasons why some motivational effects appear or not after the intervention, or why they are maintained or not over time.

### 4.1. Practical implications

Promoting PA practice from the PE setting it is not an easy task considering PE planning limitations (Casado-Robles et al., 2019). Moreover, influencing PA practice psychological mediators represents a behavior change that requires time (Neil-Sztramko et al., 2021). Results of this study suggest that planning intermittent teaching units based on activity wristbands and behavior modification strategies seems to be a more efficient distribution of the learning time than using the entire lesson for an intensive period. In this sense, achieving the PA psychological mediators change progressively during more time through intermittent teaching units, instead of concentrating all the designated time to get this target in a few weeks, could guarantee its maintenance over time. Moreover, the results obtained in the present study may guide PE teachers to design effective interventions with a different distribution of the learning time that in addition to modifying student’s PA mediators, can affect their real PA engagement, therefore reducing their high levels of physical inactivity. Additionally, the results of this research also showed that apart from influencing PA psychological mediators, other PE curricular objectives can be developed during the same lessons’ time, solving several PE planning difficulties such as the reduced time allocation to PE or the high volume of curricular contents and objectives that have to be developed during the academic course. Finally, the present study opens the way for developing potentially effective interventions applying this innovative teaching unit structure incorporating other strategies that have shown being effective for modifying PA psychological mediators in order to potentiate the outcomes obtained after the intervention. To illustrate this, as part of the intervention of other support agents like parents or tutors (Sevil-Serrano et al., 2022), and even, innovative active learning methodologies such as gamification (Arufe-Giráldez et al., 2022), which have shown being effective for improving student’s autonomous motivation, should be included.

## 5. Conclusion

An intermittent teaching unit based on the use of activity wristbands and behavior modification strategies was effective for improving studentsʼ perceptions of autonomy support and habitual PA levels. However, after six weeks of autonomous PA practice with the activity wristbands, new effects were observed in studentsʼ autonomy and relatedness needs, as well as in autonomous motivation toward PA. What is more, habitual PA levels also increase after the follow-up period. These results suggest that increasing the length of the intervention would allow to increase all psychological PA practice mediators, therefore, leading to actual PA engagement. Additionally, findings obtained in the present study and future similar studies, may help PE teachers and the scientific community to understand and design effective PA promotion programs based on behavior change theories that combine key aspects such as innovative teaching unit structures, PA self-monitoring, and behavior modification strategies that can affect student’s PA practice mediators and therefore their PA engagement.

## Data availability statement

The datasets used and analyzed during the current study are available from the corresponding author on reasonable request.

## Ethics statement

The studies involving humans were approved by Ethical Committee for Human Studies at the University of Granada (code number: 1252/CEIH/2020). The studies were conducted in accordance with the local legislation and institutional requirements. Written informed consent for participation in this study was provided by the participants’ legal guardians.

## Author contributions

SG-R, DM-V, CC-R, and JV contributed to the conception and design of the study. SG-R and CC-R were responsible for collecting the data. DM-V carried out the analysis and interpretation of the data. SG-R and JV drafted the manuscript. All authors contributed to the article and approved the final version.

## Funding

The author(s) declare financial support was received for the research, authorship, and/or publication of this article.

This publication is part of the School-Fit project (Reference number: A-SEJ-448-UGR20), funded by the FEDER/Junta de Andalucía-Consejería de Transformación Económica, Industria, Conocimiento y Universidades. [FEDER/ Regional Government of Andalusia-Ministry of Economic Transformation, Industry, Knowledge and Universities].

## Conflict of interest

The authors declare that the research was conducted in the absence of any commercial or financial relationships that could be construed as a potential conflict of interest.

## Publisher’s note

All claims expressed in this article are solely those of the authors and do not necessarily represent those of their affiliated organizations, or those of the publisher, the editors and the reviewers. Any product that may be evaluated in this article, or claim that may be made by its manufacturer, is not guaranteed or endorsed by the publisher.

## Supplementary material

The Supplementary material for this article can be found online at: https://www.frontiersin.org/articles/10.3389/fpsyg.2023.1228925/full#supplementary-material

## Footnotes

^1^ https://pulserasef.blogspot.com/

## Abbreviations

PA: Physical activity
MVPA: Moderate-to-vigorous physical activity
PE: Physical Education
SDT: Self-Determination Theory
TCM: trans-contextual model
IG: intermittent group
CG: control group
MD-PASS-PA: Multi-Dimensional Perceived Autonomy Support Scale for Physical Education
BPNES: Basic Psychological Needs in Exercise Scale
PLOC-R: Revised Perceived Locus of Causality Scale
BREQ-3: Behavioral Regulation in Exercise Questionnaire
PACE: Physician-based Assessment and Counseling for Exercise
ANOVA: one-way analyses of variance
ANCOVA: one-way analyses of covariance
