# Supplementary material for: Effect of an activity wristband-based intermittent teaching unit in Physical Education on studentsʼ physical activity and its psychological mediators: a cluster-randomized controlled trial. School-fit study
Source: Front Psychol. 2023 Sep 20;14:1228925. doi: 10.3389/fpsyg.2023.1228925 (PMC10548227; doi:10.3389/fpsyg.2023.1228925)
Supplement: Supplementary file 1 [file Table_1.DOCX]

Supplementary Material

Effect of an activity wristband-based intermittent teaching unit in Physical Education on studentsʼ physical activity and its psychological mediators: A cluster-randomized controlled trial. School-Fit study

Santiago Guijarro-Romero, Daniel Mayorga-Vega, Carolina Casado-Robles, Jesús Viciana

*** Correspondence:** Daniel Mayorga-Vega: dmayorgavega@uma.es

| Supplementary Table 1. Sensitivity analysis of the effect of the intermittent teaching unit on students’ questionnaires scores | | | | | | | | | |
| --- | --- | --- | --- | --- | --- | --- | --- | --- | --- |
|  | Pre-intervention (1) | Post-intervention (2) | Follow-up  (3) | Multilevel Linear Model^a^ | | | Effect sizes (*d*)^b^ | | |
|  | Mean (SE) | Mean (SE) | Mean (SE) | *- 2LL* | *F* | *p* | 1-2 | 2-3 | 1-3 |
| *Cognitive autonomy* |  |  |  |  |  |  |  |  |  |
| Control (*n* = 75) | 7.3 (0.2) | 7.1 (0.2) | 7.1 (0.2) | 1476.623 | 3.468 | 0.034 | 0.40 | -0.08 | 0.31 |
| Intermittent (*n* = 58) | 8.5 (0.3) | 9.1 (0.2)* | 8.9 (0.2) |  |  |  |  |  |  |
| *Procedural autonomy* |  |  |  |  |  |  |  |  |  |
| Control (*n* = 75) | 6.4 (0.2) | 6.3 (0.2) | 6.4 (0.2) | 1585.988 | 9.438 | < 0.001 | 0.72 | -0.12 | 0.60 |
| Intermittent (*n* = 58) | 7.4 (0.3) | 8.8 (0.2)*** | 8.7 (0.3)‡‡‡ |  |  |  |  |  |  |
| *Organizational autonomy* |  |  |  |  |  |  |  |  |  |
| Control (*n* = 75) | 6.9 (0.2) | 7.1 (0.2) | 7.0 (0.2) | 1497.653 | 1.866 | 0.158 | 0.25 | 0.09 | 0.34 |
| Intermittent (*n* = 58) | 8.2 (0.2) | 8.9 (0.2)* | 8.9 (0.2)‡ |  |  |  |  |  |  |
| *BPN autonomy* |  |  |  |  |  |  |  |  |  |
| Control (*n* = 75) | 7.8 (0.2) | 7.6 (0.2) | 7.7 (0.2) | 1502.690 | 6.741 | 0.002 | 0.09 | 0.46 | 0.55 |
| Intermittent (*n* = 58) | 7.8 (0.2) | 7.8 (0.2) | 8.8 (0.2)†††/‡‡‡ |  |  |  |  |  |  |
| *BPN competence* |  |  |  |  |  |  |  |  |  |
| Control (*n* = 75) | 7.8 (0.2) | 7.7 (0.2) | 7.9 (0.2) | 1569.269 | 0.662 | 0.517 | 0.06 | 0.15 | 0.20 |
| Intermittent (*n* = 58) | 8.4 (0.3) | 8.5 (0.2) | 8.9 (0.3) |  |  |  |  |  |  |
| *BPN relatedness^c^* |  |  |  |  |  |  |  |  |  |
| Control (*n* = 75) | 7.7 (0.2) | 7.7 (0.2) | 7.8 (0.2) | 1465.524 | 6.149 | 0.003 | 0.06 | 0.41 | 0.47 |
| Intermittent (*n* = 58) | 7.9 (0.2) | 8.0 (0.2) | 8.8 (0.2)†††/‡‡‡ |  |  |  |  |  |  |
| *Autonomous motivation toward PE* | |  |  |  |  |  |  |  |  |
| Control (*n* = 75) | 6.8 (0.2) | 6.9 (0.2) | 7.2 (0.2) | 1533.204 | 0.196 | 0.822 | -0.02 | 0.08 | 0.07 |
| Intermittent (*n* = 58) | 8.0 (0.3) | 8.0 (0.2) | 8.5 (0.2) |  |  |  |  |  |  |
| *Controlled motivation toward PE* | |  |  |  |  |  |  |  |  |
| Control (*n* = 75) | 4.7 (0.3) | 5.0 (0.2) | 4.9 (0.3) | 1687.400 | 3.261 | 0.041 | 0.11 | 0.38 | 0.49 |
| Intermittent (*n* = 58) | 4.6 (0.3) | 5.2 (0.3) | 5.9 (0.3)‡‡‡ |  |  |  |  |  |  |
| *Autonomous motivation toward PA^d^* | |  |  |  |  |  |  |  |  |
| Control (*n* = 75) | 7.4 (0.2) | 7.6 (0.2) | 7.4 (0.2) | 1505.386 | 8.928 | 0.003 | -0.35 | 0.57 | 0.22 |
| Intermittent (*n* = 57) | 7.9 (0.3) | 7.4 (0.2) | 8.3 (0.2)††† |  |  |  |  |  |  |
| *Controlled motivation toward PA* | |  |  |  |  |  |  |  |  |
| Control (*n* = 75) | 3.2 (0.2) | 4.2 (0.2)*** | 4.2 (0.2)‡‡‡ | 1563.864 | 6.469 | 0.002 | -0.24 | -0.42 | -0.66 |
| Intermittent (*n* = 57) | 3.7 (0.2) | 4.2 (0.3) | 3.5 (0.2)† |  |  |  |  |  |  |
| *Intention to be physically active*^d^ | |  |  |  |  |  |  |  |  |
| Control (*n* = 75) | 8.4 (0.3) | 8.1 (0.3) | 8.5 (0.2) | 1596.796 | 0.165 | 0.848 | -0.07 | -0.01 | -0.08 |
| Intermittent (*n* = 58) | 9.2 (0.3) | 8.8 (0.3) | 9.2 (0.3) |  |  |  |  |  |  |
| *Habitual PA^c^* |  |  |  |  |  |  |  |  |  |
| Control (*n* = 70) | 3.5 (0.2) | 3.8 (0.2) | 3.7 (0.2) | 1374.910 | 8.868 | < 0.001 | 0.14 | 0.44 | 0.58 |
| Intermittent (*n* = 52) | 3.5 (0.2) | 4.1 (0.2)* | 4.7 (0.2)††/‡‡‡ |  |  |  |  |  |  |
| *Note*. SE = Standard error; - 2LL = -2 log-likelihood; BPN = Basic psychological needs; PE = Physical Education; PA = Physical activity; ^a^ Multilevel Linear Model with participants nested within classes and measures nested within participants as random effects, and with the between-subjects factor *group* (control, intermittent) and the within-subject factor *time* (pre-intervention, post-intervention, follow-up) as fixed effects was selected (i.e., two-way mixed nested ANOVA/ANCOVA); *Post-hoc* within-subject pairwise comparisons with Bonferroni adjustment for each group independently: Pre-post-intervention change (* *p* < 0.05, ** *p* < 0.01, *** *p* < 0.001); Post-intervention-post-maintenance change († *p* < 0.05, †† *p* < 0.01, ††† *p* < 0.001), and pre-intervention-post-maintenance change (‡ *p* < 0.05, ‡‡ *p* < 0.01, ‡‡‡ *p* < 0.001); The covariables used for each analysis were as follows: ^c^ Gender and ^d^ Body heigh; ^b^ Cohen’s *d* effect size. | | | | | | | | | |
